# Effect of Prenatal Exposure to Lead on Estrogen Action in the Prepubertal Rat Uterus

**DOI:** 10.5402/2011/329692

**Published:** 2012-01-02

**Authors:** Andrei N. Tchernitchin, Leonardo Gaete, Rodrigo Bustamante, Aracelly Báez

**Affiliations:** Laboratory of Experimental Endocrinology and Environmental Pathology (LEEPA), Institute of Biomedical Sciences (ICBM), University of Chile Medical School, P.O. Box 21104, Santiago 21, Chile

## Abstract

Lead is a widely spread environmental pollutant known to affect both male and female reproductive systems in humans and experimental animals and causes infertility and other adverse effects. The present paper investigated the effects of prenatal exposure to lead on different parameters of estrogen stimulation in the uterus of the prepubertal rat. In prenatally and perinatally exposed rats, estrogen-induced endometrial eosinophilia, endometrial stroma edema, and eosinophil migration towards the endometrium, and uterine luminal epithelial hypertrophy are enhanced while several other responses to estrogen appear unchanged. These effects may contribute to decrease in fertility following prenatal exposure to lead. The striking difference between most of these effects of prenatal exposure and the previously reported effects of chronic exposure to lead suggests that prenatal exposure to lead may neutralize the effects of chronic exposure to lead, providing partial protection of cell function against the adverse effects of chronic exposure to lead. We propose that the mechanism involved, named imprinting or cell programming, persisted through evolution as a nongenetic adaptive mechanism to provide protection against long-term environmental variations that otherwise may cause the extinction of species not displaying this kind of adaptation.

## 1. Introduction

Infertility and other reproductive alterations may be caused by a myriad of environmental toxic agents, among them lead. This toxic environmental pollutant is widely spread and affects both male and female reproductive systems in humans [1] and in experimental animals [2]. The most known effects reported in women include infertility, increase in time needed to achieve pregnancy, miscarriage, preeclampsia, pregnancy hypertension, premature delivery [1, 3, 4], polymenorrhea, prolonged and abnormal menstruations, hypermenorrhea, and important increase in the incidence of spontaneous abortions [5]. In fact, as early as in 1965, Gilfillan [6] suggested that the declining birth rate in Rome's ruling class, which may have been at the root of the empire's dissolution, was a result of exposure to lead in food and wine. In experimental animals, chronic exposure to lead may cause an inhibition of menstruation, ovulation, and follicular growth in monkeys [7], a delay in vaginal opening in pubertal rats [8], and a decrease in frequency of implanted ova and of pregnancies in mice [9].

Several mechanisms may be involved in the alteration of fertility by lead, which were mainly investigated in experimental animals. Among them, changes may occur at the enzyme levels [10, 11] or in the action of sex steroid hormones themselves, mainly estrogens, in the uterus [12–14]. The interaction of lead with hormone action may be direct, via qualitative or quantitative changes in hormone receptors [15], or caused by changes in levels of other hormones that modify the action of sex steroids, such as glucocorticoids [16] and prolactin [17], hormones that increase under the effect of exposure to lead [18]. Further, in agreement with the existence of independent mechanisms of estrogen action in the uterus that are involved in the generation of separate groups of responses to hormone stimulation, and the report of differences in the regulation of estrogen action in each uterine cell-type [16, 19–23], it was reported that exposure to lead dissociates responses to estrogen in the uterus: it selectively enhances some of these responses, inhibits others while a third group remains unaffected [12–14].

The heterogeneity of biochemical processes related to fertility that are affected by lead, together with the existence of multiple and independent mechanisms of estrogen action [16, 19–21, 24, 25], provides an explanation for a report of time-dependent differences between the different effects of lead on reproductive changes [11]. In fact, we previously reported selective changes in some parameters of estrogen action following acute [13], subacute [12], or chronic [14] exposure to lead of prepubertal rats, which can be additionally explained in part by hematologic changes caused by lead [26] which, in turn, affect estrogen action in the uterus [16, 21, 27, 28].

Besides chronic exposure to lead, prenatal and perinatal exposure to the pollutant is one of the most common conditions affecting human population. Since the first reports linking the development of clear cell cervicovaginal adenocarcinoma in young women with diethylstilbestrol treatment of their mothers during pregnancy [29], it became clear that prenatal or neonatal exposure to several substances may additionally generate irreversible morphological, biochemical, and functional alterations that can be detected later in life. This process was named imprinting, and the first group of substances reported to cause this kind of alterations is comprised by hormones and xenobiotics displaying hormonal action [30–32]. It was suggested that the mechanism of imprinting is a modification in the path of differentiation of the affected cell types [33], which can be detected in these cells as late as in adulthood as irreversible quantitative and qualitative changes in hormone receptors or responses to hormone stimulation mediated by them [32, 34]. Based on the above mechanism, a new name was proposed for it: “cell programming (or reprogramming) process” [35, 36]. We suggested that these changes, which persist through life, facilitate the development of various diseases during the adult age [36–38]. Subsequent studies lead to the finding that not only hormones, but additionally several pharmaceutical agents, pollutants, stress, food additives, some natural components of food, and several substances present in plants, display the ability to induce imprinting [33, 35–44].

Although few studies investigated the effects of prenatal exposure to lead, several delayed effects were already reported in brain, ovary, and uterus. In the rat brain, prenatal exposure to lead was found to cause a permanent increase in the affinity of *δ*- [45] and *μ*-opioid, but not *κ*-opioid receptors [46], that parallels the impairment of opioid but not nonopioid stress-induced antinociception in developing rats [47]. In the ovary, perinatal exposure to lead was reported to cause a persistent change in LH receptors and in steroidogenesis [10], and in the uterus, lead was found to induce persistent changes in number and affinity of uterine estrogen receptors [48].

The report of delayed effects caused by prenatal exposure to lead at uterine estrogen receptor level suggests the possibility that exposure may cause persistent alterations in the action of the hormone in the uterus. The present study describes the effect of prenatal exposure to lead on estrogen action in prepubertal rat uterus, and compares these effects to those previously reported following chronic exposure to the pollutant. Additionally, the present study investigated the possible hematologic changes in rats prenatally exposed to lead, taking into consideration the role of eosinophil leukocytes in estrogen action [21].

The experimental conditions in the present study are intended to investigate, in an animal model, conditions frequently occurring in the human species: pregnant women displaying high lead blood levels as a cause of their child's prenatal exposure to lead; subsequent breast feeding as the source of the continuation of exposure after birth.

## 2. Materials and Methods

Female rats from a Sprague-Dawley-derived colony bred at the *vivarium* of the Faculty of Medicine, University of Chile, were used in the present study. Four groups of female pregnant animals were subjected to the following procedure. The animals received a single dose of lead acetate (Merck, Darmstadt, Germany) of 235 *μ*g Pb^++^ g^−1^ body wt s.c. (total lead dose averaged 61 mg Pb^++^ per animal) or saline physiological solution, at the 14th day of pregnancy (the day when spermatozoa were found in the vaginal smear was considered as the first day of pregnancy). From some lead exposed pregnant rats not included in the study of their offspring, blood samples were taken at 24, 48 72, 96, 120, and 168 h after lead injection, for measurement of their blood lead levels. Blood was also obtained from some nonexposed pregnant rats not included in the study of their offspring. The female offspring were allowed to be nursed by their exposed or nonexposed mothers up to day 21 of life, when they were treated with estradiol-17*β* (Sigma Chemical Co., St. Louis, MO, USA; 300 ng g^−1^ body wt s.c.) or its vehicle (absolute ethanol in saline physiological solution 1 : 9). The age of 21 days is the most appropriate for the study of the effects of sex steroids on target organs, since estrogen and progesterone levels are extremely low and receptor levels and hormone responsiveness are already fully developed [16]. Six or 24 h after treatment the uteri were excised under ether anesthesia, and blood samples were taken from the tail of each animal. The blood was collected into tubes containing EDTA; two samples were obtained from each animal, one was used immediately after sample collection for blood cell quantification, the other one was kept at 4°C for subsequent lead concentration determination.

The left uterine horn was fixed in 4% neutral formalin and subjected to further histological procedure for eosinophil quantification and morphometric evaluation. Paraffin sections were stained with hematoxylin-eosin as previously described [25].

Blood collected in EDTA containing tubes was used immediately after sample collection for blood cell quantification. Eosinophil quantification and evaluation of eosinophil degranulation [28] was performed in Neubauer chambers following dilution 1 : 10 with freshly prepared eosin stain solution (0.5 mL of 2% eosin Y stock solution in 100% ethanol diluted in 9 mL of distilled water and 0.5 mL of acetone). Blood leukocytes were quantified in a Neubauer camera diluting an aliquot of blood in Hayem B (0.5‰  of a saturated methylene blue solution (Merck, Darmstadt, Germany) in 3% acetic acid in distilled water) within 5 min of blood sample collection. Blood smears were fixed in methanol for 10 min and stained with May Grunwald-Giemsa (Merck, Darmstadt, Germany) for the quantification of blood leukocyte differential counts. Absolute counts were calculated with the information obtained from blood smears and Neubauer camera counts.

Blood lead concentration was measured in a group of pregnant rats nonexposed to lead (control without lead) and in another group of pregnant rats at various times after lead injection; blood lead levels were also determined in nonestrogen-treated offspring from saline or lead exposed animals, using atomic absorption spectroscopy with graphite furnace at the Chilean Institute of Public Health, Ministry of Health.

The following parameters of estrogen stimulation were investigated in the uterus: myometrial hypertrophy was measured as increase in the reciprocal value of cell density (RVCD) in circular myometrium; edema in deep endometrial stroma was evaluated as increase in RVCD in this histological location [49, 50]. Uterine eosinophilia was measured as total number of eosinophils located in the uterine horns [27] and as number of eosinophils located in each uterine histological layer. Luminal epithelial hypertrophy was evaluated as changes in cell volume [51]; measurements were performed in at least 48 cell images per animal, at locations chosen at random in the most central part of luminal epithelium for luminal epithelial cells. The cell images were obtained in a Nikon epifluorescence microscope by a cooled digital camera World Precision Instruments. The resulting images were processed by computer-assisted image analysis.

### 2.1. Statistics

Data analysis by the Tukey additivity test suggested that data on uterine eosinophils and blood lead levels should be subjected to a square root transformation to normalize distribution; the remaining data were not transformed due to their normal distribution [52]. Since multiple comparisons were performed between the 4 experimental conditions within the same time of treatment, transformed or nontransformed data were subjected to the least significant difference (LSD) test. The common variance was estimated from a one-way unbalanced analysis of variance (ANOVA) within the same time of treatment, and no significant differences were declared unless ANOVA was significant [53]. Changes in proportions of eosinophils within the different histological layers of the uterus were evaluated by the *χ* ^2^ test.

## 3. Results

### 3.1. Blood Lead Levels

Lead levels in adult pregnant rats nonexposed to lead displayed values under 2 *μ*g/dL while pregnant rats 1 to 7 days after lead exposure displayed 35.1 *μ*g/dL; no differences were detected within the 7 days following exposure. The offspring of lead-exposed mothers displayed 20.6 *μ*g/dL at the time of blood and uterus sample collection while offspring of nonexposed control mothers displayed 4.4 *μ*g/dL.

### 3.2. Hematologic Parameters

No effects of prenatal lead exposure were detected in the total number of leukocytes, eosinophils, lymphocytes, monocytes, or neutrophils per *μ*L blood in the offspring at the age of 21 or 22 days. Similarly, no changes in blood eosinophil degranulation were detected under the effect of prenatal exposure to lead (data not shown). Estrogen treatment of either exposed or nonexposed offspring did not induce any changes in these parameters.

### 3.3. Uterine Eosinophilia

**Figure 1:**
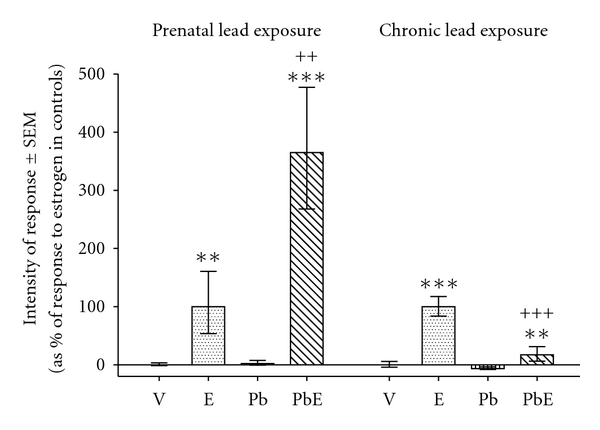
Comparison of the effects of prenatal exposure to lead and chronic exposure to lead on estrogen-induced uterine eosinophilia measured in the endometrium. Prenatally exposed rats were subjected to s.c. injection of their pregnant mothers with a lead acetate (P) or saline physiological solution at their 14th day of pregnancy. From birth on, the pups were breastfed by their mothers. At the age of 21 days the animals were treated with estradiol-17*β* (E) or vehicle (V). The uteri were obtained 6 h after hormone or vehicle administration. Previously reported data from chronically exposed rats are shown for comparison purposes (see [14]). Bars indicate means (expressed as percentage of maximal response to estradiol) ± standard error of the mean. Statistics: LSD test. ** or ^++^, *P* < 0.01; *** or ^+++^, *P* < 0.001; *, comparisons to the homologous condition without estrogen; ^+^, comparisons to the homologous condition without lead.

Prenatal exposure to lead strongly enhanced estrogen-induced increase in endometrial eosinophils at 6 h of hormone treatment (Figure 1). This response to estrogen differed from previously reported [14] decrease in endometrial eosinophils in animals under chronic exposure to lead (Figure 1). No significant change was detected in mesometrial eosinophils of estrogen-treated rats under the effect of prenatal exposure to lead (data not shown). Prenatal exposure to lead decreased the proportion of eosinophils located in the mesometrium at 6 h of hormone stimulation (from 36.8% to 20.1%; *P* < 0.001, *χ* ^2^ test). This response to estrogen in prenatally exposed animals was different from previously reported [14] increase in the proportion of eosinophils located in the mesometrium (from 38.1% to 51,9%; *P* < 0.01, *χ* ^2^ test) in animals under chronic lead exposure.

### 3.4. Endometrial Edema

**Figure 2:**
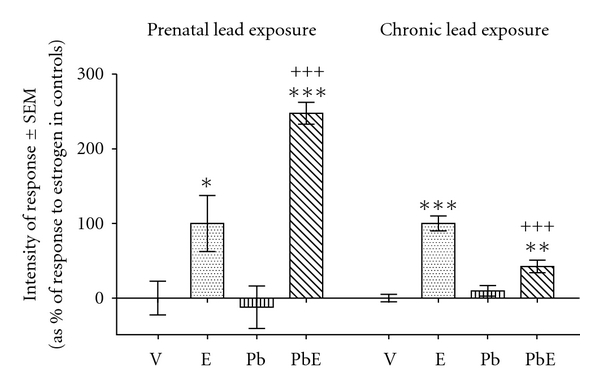
Comparison of the effects of prenatal exposure to lead and chronic exposure to lead on estrogen-induced edema in deep endometrial stroma, measured as increases in reciprocal value of cell density. Prenatally exposed rats were subjected to s.c. injection of their pregnant mothers with a lead acetate (P) or saline physiological solution at their 14th day of pregnancy. From birth on, the pups were breastfed by their mothers. At the age of 21 days the animals were treated with estradiol-17*β* (E) or vehicle (V). The uteri were obtained 6 h after hormone or vehicle administration. Previously reported data from chronically exposed rats are shown for comparison purposes (see [14]). Bars indicate means (expressed as percentage of maximal response to estradiol) ± standard error of the mean. Statistics: LSD test. *, *P* < 0.05; **, *P* < 0.01; *** or ^+++^, *P* < 0.001; *, comparisons to the homologous condition without estrogen; ^+^, comparisons to the homologous condition without lead.

Prenatal exposure to lead strongly enhanced estrogen-induced edema in deep endometrial stroma (Figure 2) at 6 h of hormone stimulation. This response differs from previously reported [14] inhibition of estrogen-induced endometrial edema following chronic exposure to lead (Figure 2).

### 3.5. Luminal Epithelial Hypertrophy

**Figure 3:**
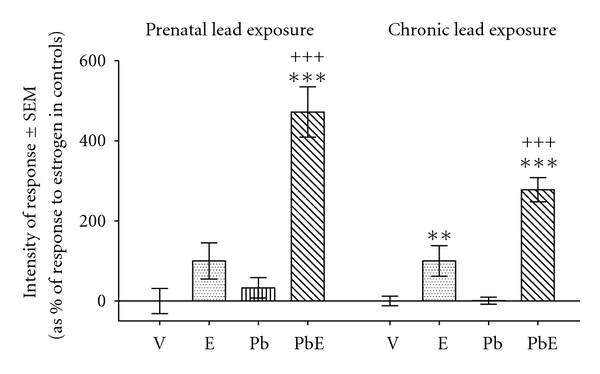
Comparison of the effects of prenatal exposure to lead and chronic exposure to lead on estrogen-induced hypertrophy of luminal epithelial cells, measured as increase in their cellular volume. Prenatally exposed rats were subjected to s.c. injection of their pregnant mothers with a lead acetate (P) or saline physiological solution at their 14th day of pregnancy. From birth on, the pups were breastfed by their mothers. At the age of 21 days the animals were treated with estradiol-17*β* (E) or vehicle (V). The uteri were obtained 24 h after hormone or vehicle administration. Previously reported data from chronically exposed rats are shown for comparison purposes (see [14]). Bars indicate means (expressed as percentage of maximal response to estradiol) ± standard error of the mean. Statistics: LSD test. **, *P* < 0.01; *** or ^+++^, *P* < 0.001; *, comparisons to the homologous condition without estrogen; ^+^, comparisons to the homologous condition without lead.

Prenatal exposure to lead strongly enhanced estrogen-induced increase in luminal epithelial cell volume 24 h after hormone stimulation (Figure 3). A previous report [14] shows a similar enhancement of estrogen-induced luminal epithelial hypertrophy under the effect of chronic exposure to lead (for comparison shown in Figure 3).

### 3.6. Hypertrophy in Circular Myometrium

**Figure 4:**
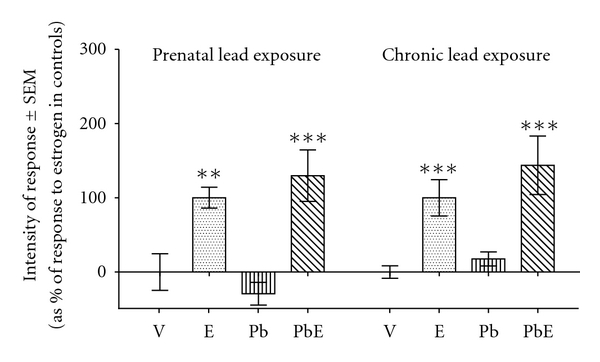
Comparison of the effects of prenatal exposure to lead and chronic exposure to lead on estrogen-induced myometrial hypertrophy, measured as increase in cell volume of circular myometrium. Prenatally exposed rats were subjected to s.c. injection of their pregnant mothers with a lead acetate (P) or saline physiological solution at their 14th day of pregnancy. From birth on, the pups were breastfed by their mothers. At the age of 21 days the animals were treated with estradiol-17*β* (E) or vehicle (V). The uteri were obtained 24 h after hormone or vehicle administration. Previously reported data from chronically exposed rats are shown for comparison purposes (see [14]). Bars indicate means (expressed as percentage of maximal response to estradiol) ± standard error of the mean. Statistics: LSD test. **, *P* < 0.01; ***P < 0.001; *, comparisons to the homologous condition without estrogen.

Lead exposure did not significantly modify estrogen-induced cell hypertrophy in this histological layer, 24 h after hormone stimulation (Figure 4). It was previously reported [14] that chronic exposure to lead neither significantly modifies this response to estrogen (for comparison shown in Figure 4).

## 4. Discussion

The present study provides evidence that prenatal exposure to lead enhances estrogen-induced uterine eosinophilia 6 h after hormone treatment, mainly in the endometrium while estrogen-induced increase in eosinophils in the mesometrium is not significantly affected. The finding of a decreased proportion of eosinophils located in the mesometrium in prenatally exposed animals suggests increased eosinophil migration from mesometrium towards myometrium and endometrium. This agrees with the increase in estrogen-induced edema in endometrial stroma in prenatally exposed animals, since endometrial edema is mediated by enzymes released from eosinophils migrating to this location; these enzymes depolymerize uterine collagen and ground substance glycosaminoglycans, increasing extravascular osmotic pressure and uterine vascular permeability [16, 21].

Several explanations may be suggested for the enhancement of estrogen-induced eosinophil migration towards endometrium in prenatally exposed rats. Eosinophils may have increased intrinsic ability for migration through connective tissue, they may contain an increased amount of enzymes needed to increase ground substance fluidity, or display increased sensitivity to chemotactic agents; the secretion of a putative eosinophil chemotactic substance by endometrial tissue [54] may be increased as well. These alterations may be mediated by qualitative or quantitative changes in estrogen receptors, described to occur following prenatal exposure to lead [48], or by alterations in regulatory mechanisms. The latter may occur in the affected uterine cell types and include proteins such as heat shock proteins, for instance hsp90, which is known to interact with estrogen receptors and modify their activity [55], or hsp70, which also bind estrogen receptors and may protect them against a number of adverse conditions [56, 57]. It may also involve systemic endocrine changes, such as glucocorticoids, catecholamines, prolactin or growth hormone [18], which are known to modify responses to estrogen [16, 17]; these possibilities need to be further investigated.

The present report describes an important enhancement of estrogen-induced uterine luminal epithelium hypertrophy under the effect of prenatal exposure to lead. It is similar to that reported following acute [13], subacute [12], or chronic [14] exposure to lead. In contraposition to findings in the uterine luminal epithelium, prenatal exposure did not cause a significant change in estrogen-induced hypertrophy in circular myometrium, an effect that was not found following chronic exposure either [14]. The difference between both cell types may be explained by the accumulation of lead in luminal epithelium [58], or to differences in sensitivity of these cell-types to lead exposure. This finding confirms our previous suggestions that toxic substances may interact in a different way with the different uterine cell-types [12–14, 16], and points to the need for the consideration of all cell types in every study of toxicity in reproductive organs.

With regard to hematologic parameters reported to be affected following subacute [59] or chronic [26] exposure to lead (increase in the degranulation of blood eosinophils and increase in total leukocyte counts, eosinophils, lymphocytes, monocytes, and neutrophils in the blood), prenatal exposure to lead did not revealed any delayed effects on them. Taking into consideration the role of eosinophils in estrogen action in the uterus, the lack of effect of exposure on these leukocytes may explain in part differences in responses to estrogen in the uterus following the different times of exposure to lead.

It is striking that three responses to estrogen stimulation (estrogen-induced endometrial eosinophilia, eosinophil migration from mesometrium towards myometrium and endometrium, and endometrial edema), but not the remaining responses (luminal epithelial and myometrial cell hypertrophy), display just the opposite behavior when comparing prenatal exposure and chronic exposure. Estrogen-induced increase in eosinophils in the endometrium appears to be enhanced in prenatally exposed rats while it is inhibited following chronic exposure. Eosinophil migration from mesometrium towards myometrium and endometrium is enhanced in prenatally exposed animals while it is inhibited in chronically exposed rats. Estrogen-induced endometrial edema is enhanced in prenatally exposed rats while the response is inhibited following chronic exposure. The differences appears even more conspicuously, considering that lead blood levels in prenatally exposed rats at the age of estrogen stimulation are much higher than those in nonexposed controls, although lower than following chronic exposure, that is, mimicking a less severe chronic exposure.

Since it was reasonable to expect, in prenatally exposed rats, an inhibition of endometrial eosinophilia and edema of a smaller magnitude than in prenatally exposed rats, our finding of enhancement of the responses to estrogen in prenatally exposed animals was completely unexpected. Our finding suggests that prenatal exposure may antagonize the effects of chronic exposure to lead in endometrial eosinophilia and edema, thus providing a partial protection of cell function against adverse effects of chronic exposure to lead.

We have previously proposed that estrogen-induced endometrial edema and destruction of endometrial extracellular matrix by plasmin formed by eosinophil plasminogen activators are required to facilitate blastocyst implantation [16]. Eosinophils were also proposed to suppress some immune reaction sequelae that could affect the development of the product of conception [16]. Therefore, if the inhibition of estrogen-induced endometrial eosinophilia and edema under chronic exposure to lead contribute to the infertile condition, prenatal exposure may neutralize these effects and provide a partial protection against these adverse effects of chronic exposure.

Nevertheless, at this point, it is not possible to ascertain whether the remaining effects of prenatal exposure to lead in the uterus may interfere with blastocyst implantation and its development in the uterus. Further work is needed to evaluate this possibility as well as effects in other organs of the reproductive system.

Imprinting can be considered a general biological epigenetic mechanism. It causes changes in the differentiation or programming in various cell-types under the effect of perinatal exposure to various agents or conditions; these changes persist through life and can be detected through biochemical, morphological, and functional changes in affected cells. We now propose that this process may have persisted through evolution as a non-genetic adaptive mechanism to provide protection against long-term environmental variations that otherwise may cause the extinction of species not displaying this kind of adaptation. In the particular case of lead, it may protect several reproductive functions and their mechanisms against damage by this pollutant, allowing offspring of prenatally or perinatally exposed individuals to survive and further reproduce in a newly polluted environment.

Lead is also known to affect other organs and systems. For instance, it causes neurological and neurobehavioral changes that were reported in countries with high lead pollution levels [60–63]. Among these changes, it impairs learning in experimental animals [64] and in humans [63, 65], and development of aggressive and delinquent behavior [66] that evolved in correlation with lead blood levels during preschool ages in different countries [67]. Studies in rats had shown that chronic exposure to lead in rats induces astroglial changes in the brain and reduces dopaminergic neurons in the substantia nigra [68], increases brain serotonin as well as immunoreactive serotoninergic cell bodies density in the dorsal raphe nucleus, and increases in anxiety [69]. Prenatal exposure to lead, in turn, affected in the postnatal age, energy status of neuronal mitochondria, and altered neuronal function in such a way that could play a role in neurodegeneration [70]. No studies were reported comparing the effects of chronic exposure and of prenatal exposure on the same neurologic and neurobehavior parameters. These studies are necessary to ascertain whether there is an antagonism of prenatal exposure on chronic exposure effects in a similar way as described for the uterus in the present report.

## 5. Conclusion

We conclude from this study that prenatal exposure to lead causes persistent changes in several responses to estrogen in the rat uterus, detected at postnatal age of 21 days, and suggest that prenatal exposure may constitute a non-genetic adaptive mechanism to antagonize some of the adverse effects of chronic exposure to lead in the uterus, thus protecting it against reproductive impairment caused by chronic exposure to lead.

## References

[1] Winder C (1993). Lead, reproduction and development. *NeuroToxicology*.

[2] Ronis MJJ, Badger TM, Shema SJ, Roberson PK, Shaikh F (1996). Reproductive toxicity and growth effects in rats exposed to lead at different periods during development. *Toxicology and Applied Pharmacology*.

[3] Guerra-Tamayo JL, Hernández-Cadena L, Tellez-Rojo MM (2003). Tiempo para el embarazo y exposición a plomo. *Salud Pública de México*.

[4] Al-Saleh I, Coskun S, Mashhour A (2008). Exposure to heavy metals (lead, cadmium and mercury) and its effect on the outcome of in-vitro fertilization treatment. *International Journal of Hygiene and Environmental Health*.

[5] Tang N, Zhu ZQ (2003). Adverse reproductive effects in female workers of lead battery plants. *International Journal of Occupational Medicine and Environmental Health*.

[6] Gilfillan SC (1965). Lead poisoning and the fall of Rome. *Journal of Occupational Medicine*.

[7] Eck GJVV, Meigs JW (1960). Changes in the ovary of the Rhesus monkey after chronic lead intoxication. *Fertility and Sterility*.

[8] Kimmel CA, Grant LD, Sloan CS, Gladen BC (1980). Chronic low-level lead toxicity in the rat. I. Maternal toxicity and perinatal effects. *Toxicology and Applied Pharmacology*.

[9] Odenbro A, Kihlstrom JE (1977). Frequency of pregnancy and ova implantation in triethyl lead treated mice. *Toxicology and Applied Pharmacology*.

[10] Wiebe JP, Barr KJ, Buckingham KD (1988). Effect of prenatal and neonatal exposure to lead on gonadotropin receptors and steroidogenesis in rat ovaries. *Journal of Toxicology and Environmental Health*.

[11] Kempinas WG, Favaretto ALV, Melo VR, Carvalho TLL, Petenusci SO, Oliveira-Filho RM (1994). Time-dependent effects of lead on rat reproductive functions. *Journal of Applied Toxicology*.

[12] Tchernitchin NN, Tchernitchin AN, Mena MA, Villarroel C, Guzmán C, Poloni P (1998). Effect of subacute exposure to lead on responses to estrogen in the immature rat uterus. *Bulletin of Environmental Contamination and Toxicology*.

[13] Tchernitchin NN, Villagra A, Tchernitchin AN (1998). Antiestrogenic activity of lead. *Environmental Toxicology and Water Quality*.

[14] Tchernitchin NN, Clavero A, Mena MA (2003). Effect of chronic exposure to lead on estrogen action in the prepubertal rat uterus. *Environmental Toxicology*.

[15] Wide M, Wide L (1980). Estradiol receptor activity in uteri of pregnant mice given lead before implantation. *Fertility and Sterility*.

[16] Tchernitchin AN, Mena MA, Rodríguez A, Maturana M, Pertschuk LP, Lee SH (1985). Radioautographic localization of estrogen receptors in the rat uterus: a tool for the study of classical and nontraditional mechanism of hormone action. *Localization of Putative Steroid Receptors*.

[17] Unda C, Arriagada R, Ramírez L, Vásquez MV, Tchernitchin AN (1989). Adenohypophysis reimplants selectively block a genomic response to estrogen in the immature rat uterus. *Medical Science Research*.

[18] Vyskocil A, Fiala Z, Tejnorová I, Tusl M (1991). Stress reaction in developing rats exposed to 1% lead acetate. *Sbornik Vedeckych Praci Lekarske Fakulty Karlovy University v Hradci Kralove*.

[19] Tchernitchin A (1979). The role of eosinophil receptors in the non-genomic response to estrogens in the uterus. *Journal of Steroid Biochemistry*.

[20] Tchernitchin AN (1983). Eosinophil-mediated non-genomic parameters of estrogen stimulation–a separate group of responses mediated by an independent mechanism. *Journal of Steroid Biochemistry*.

[21] Tchernitchin AN, Mena MA, Soto J, Unda C (1989). The role of eosinophils in the action of estrogens and other hormones. *Medical Science Research*.

[22] Gaete L, Tchernitchin AN, Bustamante R (2010). Biological activity of genistein and soy extracts: selective induction of some but not all estrogenic responses in the prepubertal rat uterus. *Boletín Latinoamericano y del Caribe de Plantas Medicinales y Aromáticas*.

[23] Gaete L, Tchernitchin AN, Bustamante R (2011). Genistein selectively inhibits estrogen-induced cell proliferation and some other responses to hormone stimulation in the prepubertal rat uterus. *Journal of Medicinal Food*.

[24] Tchernitchin AN, Galand P (1982). Dissociation of separate mechanisms of estrogen action by actinomycin D. *Experientia*.

[25] Tchernitchin AN, Galand P (1983). Oestrogen levels in the blood, not in the uterus, determine uterine eosinophilia and oedema. *Journal of Endocrinology*.

[26] Tchernitchin AN, Villagra R, Tchernitchin NN (1997). Effect of chronic exposure to lead on immature rat leucocytes. *Medical Science Research*.

[27] Tchernitchin A, Roorijck J, Tchernitchin X, Vandenhende J, Galand P (1974). Dramatic early increase in uterine eosinophils after oestrogen administration. *Nature*.

[28] Tchernitchin AN, Barbera J, Arroyo P, Mena MA, Vilches K, Grunert G (1985). Degranulatory action of estradiol on blood eosinophil leukocytes in vivo and in vitro. *Agents and Actions*.

[29] Herbst AL (1981). Clear cell adenocarcinoma and the current status of DES-exposed females. *Cancer*.

[30] Csaba G, Nagy SU (1976). Plasticity of the hormone receptors and possibility of their deformation in neonatal age. *Experientia*.

[31] Csaba G (1980). Phylogeny and ontogeny of hormone receptors: the selection theory of receptor formation and hormonal imprinting. *Biological Reviews of the Cambridge Philosophical Society*.

[32] Csaba G, Inczefi-Gonda A, Dobozy O (1986). Hormonal imprinting by steroids: a single neonatal treatment with diethylstilbestrol or allylestrenol gives rise to a lasting decrease in the number of rat uterine receptors. *Acta Physiologica Hungarica*.

[33] Tchernitchin AN, Tchernitchin N (1992). Imprinting of paths of heterodifferentiation by prenatal or neonatal exposure to hormones, pharmaceuticals, pollutants and other agents and conditions. *Medical Science Research*.

[34] Dobozy O, Csaba G, Hetenyi G, Shahin M (1985). Investigation of gonadotropin-thyrotropin overlapping and hormonal imprinting in the rat testis. *Acta Physiologica Hungarica*.

[35] Tchernitchin AN, Tchernitchin NN, Mena MA, Unda C, Soto J (1999). Imprinting: perinatal exposures cause the development of diseases during the adult age. *Acta Biologica Hungarica*.

[36] Tchernitchin AN (2005). Perinatal exposure to chemical agents: delayed effects by the mechanism of imprinting (cell programming). *Annual Review of Biomedical Sciences*.

[37] Tchernitchin AN, Mena MA (2006). Efectos diferidos de contaminantes ambientales y otros agentes en salud reproductiva y sexualidad: un desafío pendiente de la toxicología de la reproducción para la salud de las futuras generaciones. *Cuadernos Médicos Sociales*.

[38] Tchernitchin AN, Bustamante R, Gaete L (2009). Exposición prenatal a drogas de abuso. Efectos diferidos e imprinting (desprogramación celular). *Cuadernos Médico Sociales*.

[39] Tchernitchin AN, Tchernitchin NN (1999). Perinatal exposure to substances present in plants and other compounds causes the development of diseases during the adult age, by the mechanism of imprinting. *Acta Horticulture*.

[40] Adewale HB, Todda KL, Mickens JA, Patisaul HB (2011). The impact of neonatal bisphenol-A exposure on sexually dimorphic hypothalamic nuclei in the female rat. *NeuroToxicology*.

[41] Dickerson SM, Cunningham SL, Patisaul HB, Woller MJ, Gore AC (2011). Endocrine disruption of brain sexual differentiation by developmental PCB exposure. *Endocrinology*.

[42] Somm E, Schwitzgebel VM, Toulotte A (2009). Perinatal exposure to bisphenol A alters early adipogenesis in the rat. *Environmental Health Perspectives*.

[43] Vreugdenhil HJI, Slijper FME, Mulder PGH, Weisglas-Kuperus N (2002). Effects of perinatal exposure to PCBs and dioxins on play behavior in Dutch children at school age. *Environmental Health Perspectives*.

[44] Gunawardana L, Zammit S, Lewis G (2011). Examining the association between maternal analgesic use during pregnancy and risk of psychotic symptoms during adolescence. *Schizophrenia Research*.

[45] McDowell J, Kitchen I (1988). Perinatal lead exposure alters the development of *δ*- but not *μ*-opioid receptors in rat brain. *British Journal of Pharmacology*.

[46] Kitchen I (1993). Lead toxicity and alterations in opioid systems. *NeuroToxicology*.

[47] Jackson HC, Kitchen I (1989). Perinatal lead exposure impairs opioid but not non-opioid stress-induced antinociception in developing rats. *British Journal of Pharmacology*.

[48] Wiebe JP, Barr KJ (1988). Effect of prenatal and neonatal exposure to lead on the affinity and number of estradiol receptors in the uterus. *Journal of Toxicology and Environmental Health*.

[49] Grunert G, Fernandez S, Tchernitchin AN (1984). Methods for the evaluation of responses to estrogen in individual cell types or regions of the uterus. *Hormone Research*.

[50] Grunert G, Porcia M, Tchernitchin AN (1986). Differential potency of oestradiol-17*β* and diethylstilboestrol on separate groups of responses in the rat uterus. *Journal of Endocrinology*.

[51] Tchernitchin AN, Kattan F, Tchernitchin NN (1995). Dose-response of estradiol-17*β* on uterine luminal and glandular epithelial hypertrophy, evaluated morphometrically. *Medical Science Research*.

[52] Grunert G, Porcia M, Neumann G, Sepúlveda S, Tchernitchin AN (1984). Progesterone interaction with eosinophils and with responses already induced by oestrogen in the uterus. *Journal of Endocrinology*.

[53] Snedecor GW, Cochran WG (1967). *Statistical Methods*.

[54] Lee YH, Howe RS, Sha SJ, Teuscher C, Sheehan DM, Lyttle CR (1989). Estrogen regulation of an eosinophil chemotactic factor in the immature rat uterus. *Endocrinology*.

[55] Knoblauch R, Garabedian MJ (1999). Role for Hsp90-associated cochaperone p23 in estrogen receptor signal transduction. *Molecular and Cellular Biology*.

[56] Klinge CM, Brolly CL, Bambara RA, Hilf R (1997). Hsp70 is not required for high affinity binding of purified calf uterine estrogen receptor to estrogen response element DNA in vitro. *Journal of Steroid Biochemistry and Molecular Biology*.

[57] Ciocca DR, Green S, Elledge RM (1998). Heat shock proteins hsp27 and hsp70: lack of correlation with response to tamoxifen and clinical course of disease in estrogen receptor-positive metastatic breast cancer (a Southwest Oncology Group study). *Clinical Cancer Research*.

[58] Nilsson BO, Ljung L, Wide M (1991). Electron microscopy and X-ray microanalyses of uterine epithelium from lead-injected mice in an experimental delay of implantation. *Archives of Toxicology*.

[59] Villagra R, Tchernitchin NN, Tchernitchin AN (1997). Effect of subacute exposure to lead and estrogen on immature pre- weaning rat leukocytes. *Bulletin of Environmental Contamination and Toxicology*.

[60] Tchernitchin AN, Lapin N, Molina L (2005). Human exposure to lead in Chile. *Reviews of Environmental Contamination and Toxicology*.

[61] Laidlaw MAS, Filippelli GM (2008). Resuspension of urban soils as a persistent source of lead poisoning in children: a review and new directions. *Applied Geochemistry*.

[62] Donaldson SG, van Oostdam J, Tikhonov C (2010). Environmental contaminants and human health in the Canadian Arctic. *Science of the Total Environment*.

[63] Mielke HW, Laidlaw MAS, Gonzales CR (2011). Estimation of leaded (Pb) gasoline’s continuing material and health impacts on 90 US urbanized areas. *Environment International*.

[64] Massaro TF, Miller GD, Massaro EJ (1986). Low-level lead exposure affects latent learning in the rat. *Neurobehavioral Toxicology and Teratology*.

[65] Needleman HL, Schell A, Bellinger D, Leviton A, Allred EN (1990). The long-term effects of exposure to low doses of lead in childhood. An 11-year follow-up report. *The New England Journal of Medicine*.

[66] Needleman HL, Riess JA, Tobin MJ, Biesecker GE, Greenhouse JB (1996). Bone lead levels and delinquent behavior. *Journal of the American Medical Association*.

[67] Nevin R (2007). Understanding international crime trends: the legacy of preschool lead exposure. *Environmental Research*.

[68] Sansar W, Ahboucha S, Gamrani H (2011). Chronic lead intoxication affects glial and neural systems and induces hypoactivity in adult rat. *Acta Histochemica*.

[69] Sansar W, Bouyatas MM, Ahboucha S, Gamrani H (2012). Effects of chronic lead intoxication on rat serotoninergic system and anxiety behavior. *Acta Histochemica*.

[70] Baranowska-Bosiacka I, Gutowska I, Marchetti C (2011). Altered energy status of primary cerebellar granule neuronal cultures from rats exposed to lead in the pre- and neonatal period. *Toxicology*.

